# Selection of superior accessions of turnip (*Brassica rapa* var. *rapa* L.) based on tuber quality‐related characters

**DOI:** 10.1002/fsn3.2871

**Published:** 2022-04-01

**Authors:** Ali Khadivi, Farhad Mirheidari, Younes Moradi

**Affiliations:** ^1^ 125649 Department of Horticultural Sciences Faculty of Agriculture and Natural Resources Arak University Arak Iran

**Keywords:** *Brassica rapa* var. *rapa*, breeding, conservation, diversity, gene pool

## Abstract

Turnip (*Brassica rapa* var. *rapa* L.) (syn. *B. campestris* L. ssp. *rapifera* Sinsk) is an important crop species belonging to the Brassicaceae family. The 185 accessions belonging to this crop were collected from several areas of Toodshak region in Isfahan province, Iran, and their tubers were cultivated under homogeneous conditions in loamy clay soil. The morphological traits of different organs of those accessions were evaluated. Significant variations were detected among the accessions studied based on the traits recorded. Tuber shape showed high variation and included globose, oblong, ovate, obovate, and fusiform. Also, tuber skin color was highly variable, including white, bicolor white–violet, light violet, and dark violet. Tuber weight ranged from 1.56 to 35.70 g, while total soluble solids (TSS) of tuber flesh ranged from 7.00 to 11.80%. Principal component analysis (PCA) showed that 18 components were extracted by explaining 74.88% of total variance. The dendrogram obtained based on all the characters measured clustered the accessions into two major clusters. Sixteen accessions were placed into the first cluster, while the remaining accessions were placed into the second cluster which was divided into six subclusters. High level of morphological variabilities was observed among the accessions, which is applicable and useful for *B. rapa* var. *rapa* breeding programs. Based on the commercial and quality traits, 17 accessions could be selected for direct cultivation. Also, the promising accessions identified here can be utilized directly in breeding programs for genetic enhancement of this crop.

## Graphical abstract

Based on the commercial and quality traits, 17 accessions of turnip (*Brassica rapa* var. *rapa* L.) could be selected for direct cultivation. Also, the promising accessions identified here can be utilized directly in breeding programs for genetic enhancement of this crop. Tuber skin color was highly variable, including white, bicolor white–violet, light violet, and dark violet. Tuber weight ranged from 1.56 to 35.70 g. Tuber flesh TSS ranged from 7.00 to 11.80%. Principal component analysis (PCA) showed that 18 components were extracted by explaining 74.88% of total variance.

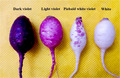

## 1 INTRODUCTION

Turnip (*Brassica rapa* var. *rapa* L.) (syn. *B. campestris* L. ssp. *rapifera* Sinsk) is an important crop species belonging to the Brassicaceae family. It is an annual or biennial plant with wide variation in size, shape, and color. Its classification is based on morphological characteristics, leading to a division of the cultivated forms into three main subspecies: turnip, oilseed, and leafy types. Thus, it plays a vital role in agriculture, and contributes to both national economies and human health. The *B. rapa* var. *rapa* is a vital cruciferous vegetable species, with unique physiology and morphology traits. Its plant parts, including both roots and leaves, are important for human consumption and also for animal feed (Bonnema et al., 2019; Chung et al., 2016; Liu et al., 2019; Lou et al., 2017). The main edible organ is consisted of the swollen hypocotyl and root (Kubo et al., 2019). It is generally grown as a field vegetable or home garden crop (Abbasi et al., 2011). Turnip is greatly consumed in Europe, Asia, America, and North Africa due to its availability in local markets, cheapness, and consumer preference (Haliloglu et al., 2012; Li et al., 2018). This species is frequently grown in temperate climatic region (Javed et al., 2019; Lu et al., 2008; Saeed et al., 2012).

Genetic diversity is a prerequisite in crop improvement programs. It aids in choosing parental materials to get maximum recombination in hybridization process (Arunachalam, 1981). Diversity in germplasm is fundamental in selecting superior accessions having advantageous traits for utilization in hybridization in developing varieties with increased yield (Joshi & Dhawan, 1966) and wider adaptation, desirable quality pest, and disease resistance (Nevo et al., 1982). Besides genetic variability in the germplasm, the effectiveness of selection for a particular trait depends on the degree of association that exists between different traits.

Analysis of variability among the traits and the association of a particular character in relation to other traits contributing to yield of a crop would be of great importance in planning a successful breeding program (Mary & Gopalan, 2006). The main aim of plant breeders is to study the genetic variability of new accessions of important crop species and to screen best accessions for efficient utilization or varieties development. Estimation of various qualitative and quantitative traits provides a clear picture to improve diverse accessions. Among these characters, the yield parameter plays a vital role for new variety development (Ali et al., 2013; Azam et al., 2013).

Morphological characterization continues to be the first step in the description and classification of germplasm (Marjanovic‐Jeromela et al., 2011). Cluster and principal component analyses are useful techniques to be used for the classification of different biological populations at genotypic level and to evaluate comparative influence of various components to the total divergence both at intra‐ and intercluster levels. In plant breeding programs, several characters are simultaneously considered making it feasible to approximate the genetic divergence using multivariate techniques. Multivariate analyses have equivalent usefulness to create the most proper cross combinations (Zahan et al., 2008).

Collections of local landraces/germplasm from diverse locations have great value from breeding point of view as containing many hidden desirable genes. For a successful plant breeding program, it is necessary that germplasm have diversity, reproducibility, and easily availability to be used in the development of new cultivars (Naushad et al., 2015; Zada et al., 2013). The present study attempted to study agro‐morphological‐based variation among different local accessions of *B. rapa* var. *rapa* for economically important quantitative traits.

## 2 MATERIAL AND METHODS

### 2.1 Plant material

The 185 accessions belonging to *B. rapa* var. *rapa* were collected from several areas of Toodshak region in Isfahan province, Iran. The tubers of the collected accessions were cultivated in Saveh region, Markazi province, Iran, under homogeneous conditions in loamy clay soil.

### 2.2 The characters evaluated

In total, 63 morphological characters were used to study phenotypic diversity (Table 1). A digital caliper was used to measure dimensions‐related characters of leaf, flower, tuber, silique, and seed. Also, tuber weight, silique weight, and 1000‐seed weight were measured with an electronic balance with 0.01 g precision. Furthermore, the qualitative characters were estimated based on rating and coding (Table 2).

**TABLE 1:** Statistical descriptive parameters for morphological traits used to study *B. rapa* var. *rapa* accessions

No.	Character	Unit	Min.	Max.	Mean	SD	CV (%)
1	Tuber shape	Code	1	9	5.11	2.90	56.65
2	Tuber skin color	Code	1	7	3.98	2.28	57.19
3	Tuber weight	g	1.56	35.70	10.10	6.64	65.79
4	Tuber surface	Code	1	5	3.02	1.52	50.26
5	Tuber length	mm	21.80	59.15	37.61	6.69	17.78
6	Tuber middle diameter	mm	12.36	48.24	26.76	6.93	25.88
7	Tuber maximum transverse diameter	mm	14.20	54.63	29.11	7.35	25.26
8	Tuber maximum transverse diameter position	Code	1	5	3.08	1.26	40.81
9	Tuber collar diameter (neck)	mm	4.79	16.86	9.09	2.22	24.38
10	Tuber tail length	mm	51.76	115.22	86.47	14.86	17.18
11	Tuber flesh bitterness	Code	0	3	0.12	0.41	344.17
12	Tuber flesh texture	Code	1	5	4.58	0.92	20.04
13	Tuber flesh TSS	%	7.00	11.80	9.39	0.98	10.44
14	Tuber main shoot number	Number	1	4	1.14	0.40	35.18
15	Leaf length	mm	53.00	173.20	113.24	26.73	23.61
16	Petiole length	mm	16.95	106.12	59.85	21.24	35.48
17	Petiole width	mm	1.16	4.98	2.76	0.49	17.81
18	Clasping of stem by upper leaves	Code	1	3	2.72	0.70	25.63
19	Cauline leaves color	Code	1	3	1.83	0.99	54.04
20	Floral leaves color	Code	1	7	4.05	1.22	30.05
21	Lamina length (lamina blade length)	mm	28.30	99.10	54.28	15.58	28.70
22	Lamina width (lamina blade width)	mm	21.06	63.20	40.57	9.59	23.65
23	Leaf apex shape	Code	1	7	4.47	1.34	29.87
24	Leaf blade shape outline (lamina)	Code	1	13	7.15	4.44	62.10
25	Leaf lamina attitude	Code	1	5	3.52	1.08	30.65
26	Leaf lamina blistering	Code	0	3	0.22	0.58	262.27
27	Leaf division	Code	1	5	4.10	1.43	34.83
28	Leaf margin	Code	1	11	5.31	2.87	54.01
29	Leaf lobelet number	Number	0	8	2.42	1.97	81.24
30	Lobelet arrangement	Code	0	3	1.24	1.15	92.90
31	Flowering date	Date	1	5	3.16	0.69	21.77
32	Plant height until first flower	mm	95.00	273.00	148.45	26.48	17.84
33	Total plant height	mm	325.00	823.00	541.94	111.72	20.61
34	Flowering branch number	Number	5	39	15.17	5.80	38.22
35	Leaf number until first flower	Number	5	20	11.41	2.70	23.64
36	Total leaf number	Number	10	75	33.52	10.02	29.90
37	Flower length	mm	10.75	18.62	14.69	1.50	10.18
38	Flower width	mm	7.80	15.21	11.17	1.43	12.82
39	Petal length	mm	4.95	13.14	7.12	0.96	13.44
40	Petal width	mm	3.29	5.83	4.68	0.53	11.22
41	Seed ripening time	Date	1	5	2.35	1.61	68.30
42	Silique number in main stem	Number	2	39	13.05	6.91	52.91
43	Total silique number in plant	Number	5	820	108.91	97.30	89.34
44	Silique length	mm	24.45	59.17	41.38	6.76	16.34
45	Silique width	mm	2.43	6.05	3.84	0.70	18.21
46	Silique thickness	mm	1.67	5.15	2.78	0.48	17.31
47	Silique surface outline	Code	1	5	2.57	1.87	72.88
48	Silique shape	Code	1	9	5.49	2.61	47.60
49	Dry silique color	Code	1	9	4.92	2.82	57.40
50	Silique shattering	Code	0	5	1.59	1.48	93.08
51	Silique beak length	mm	3.47	31.25	13.04	4.24	32.54
52	Silique pedicel length	mm	5.61	38.66	17.06	3.81	22.36
53	Distinct silique pedicel node color	Code	0	1	0.84	0.37	44.05
54	Silique pedicel node color	Code	1	13	7.09	3.46	48.76
55	Silique weight	g	0.02	0.11	0.05	0.02	31.89
56	Silique total seed number (rounded +wrinkled)	Number	10	54	23.55	7.47	31.71
57	Silique seed number (rounded)	Number	0	43	17.41	8.19	47.02
58	Seed color	Code	1	5	3.09	1.57	50.87
59	Seed length	mm	1.01	2.29	1.57	0.23	14.39
60	Seed width	mm	0.69	1.78	1.26	0.20	15.63
61	1000‐seed weight	g	0.00	2.16	1.22	0.34	27.99
62	Vivipary	Code	0	1	0.16	0.37	228.13
63	Aerial part dry weight	g	3.20	252.20	38.10	37.56	98.58

**TABLE 2:** Frequency distribution for the measured qualitative morphological characters in the studied *B. rapa* var. *rapa* accessions

Character	Frequency (no. of accessions)							
	0	1	3	5	7	9	11	13
Tuber shape	‐	Globose (38)	Oblong (34)	Ovate (33)	Obovate (40)	Fusiform (40)	‐	‐
Tuber skin color	‐	White (50)	Bicolor white–violet (40)	Light violet (49)	Dark violet (46)	‐	‐	‐
Tuber surface	‐	Smooth (52)	A bit wrinkled (79)	Very wrinkled (54)	‐	‐	‐	‐
Tuber maximum transverse diameter position	‐	Toward base (33)	Central (112)	Towards apex (40)	‐	‐	‐	‐
Tuber flesh bitterness	Absent (167)	Low (16)	Intermediate (2)	‐	‐	‐	‐	‐
Tuber flesh texture	‐	Soft (4)	Slightly soft (31)	Firm (150)	‐	‐	‐	‐
Clasping of stem by upper leaves	‐	Approx. 50% (26)	Approx. 100% (159)		‐	‐	‐	‐
Cauline leaves color	‐	Light green (108)	Green (77)	‐	‐	‐	‐	‐
Floral leaves color	‐	Light green (4)	Green (87)	Dark green (87)	Green–silver (7)	‐	‐	‐
Leaf apex shape	‐	Acute (4)	Intermediate (60)	Rounded (102)	Broadly rounded (19)	‐	‐	‐
Leaf blade shape outline (lamina)	‐	Deltoid (42)	Orbicular (7)	Elliptic (36)	Obovate (17)	Spathulate (7)	Ovate (45)	Oblong (31)
Leaf lamina attitude	‐	Down curling (9)	Flat (119)	Up curling (57)	‐	‐	‐	‐
Leaf lamina blistering	None (155)	Low (25)	Intermediate (5)		‐	‐	‐	‐
Leaf division	‐	Entire (24)	Sinuate (35)	Lyrate (126)	‐	‐	‐	‐
Leaf margin	‐	Crenate (37)	Broadly crenate (13)	Sinuate (57)	Broadly sinuate (55)	Serrate (8)	Broadly serrate (15)	
Lobelet arrangement	Absent (55)	Opposite (80)	Alternate (50)	‐	‐	‐	‐	‐
Flowering date	‐	Late March (4)	Early April (162)	Mid‐April (19)	‐	‐	‐	‐
Seed ripening time	‐	Late May (99)	Early June (47)	Mid‐June (39)	‐	‐	‐	‐
Silique surface outline	‐	Smooth (105)	Undulating (55)	Constricted between seeds (65)				‐
Silique shape	‐	Cylindrical (41)	Spathulate (5)	Oblong (19)	Lanceolate (108)	Deltoid (12)	‐	‐
Dry silique color	‐	Light golden yellow (49)	Golden yellow (25)	Cream‐light brown (10)	Light brown (86)	Brown (15)	‐	‐
Silique shattering	None (43)	Low (81)	Intermediate (46)	High (15)	‐	‐	‐	‐
Distinct silique pedicel node color	No (30)	Yes (155)	‐	‐	‐	‐	‐	‐
Silique pedicel node color	‐	Homochromatic (30)	Light brown (11)	Brown (4)	Light grey (59)	Grey (49)	Dark grey (21)	Black (11)
Seed color	‐	Light brown (53)	Brown (71)	Dark brown (61)	‐	‐	‐	‐
Vivipary	No (156)	Yes (29)	‐	‐	‐	‐	‐	‐

### 2.3 Statistical analysis

Analysis of variance (ANOVA) was performed to evaluate the variation among accessions based on the traits measured using SAS software (SAS Institute, 1990). Simple correlations between traits were determined using Pearson correlation coefficients (SPSS Inc., Norusis, 1998). Principal component analysis (PCA) was used to investigate the relationship between accessions and determine the main traits effective in genotype segregation using SPSS software. Hierarchical cluster analysis (HCA) was performed using Ward's method and Euclidean coefficient using PAST software (Hammer et al., 2001). The first and second principal components (PC1/PC2) were used to create a scatter plot with PAST software.

## 3 RESULTS AND DISCUSSION

Significant variations were detected among the accessions studied based on the traits recorded as revealed with ANOVA. Forty‐nine of 63 characters measured showed CV more than 20.00%, 20 characters showed the CV more than 50.00%, and three characters exhibited the CV more than 100.00%, indicating high diversity among the accessions. Tuber flesh bitterness had the highest CV (344.17%), followed by leaf lamina blistering (262.27%) and vivipary (228.13%). The lowest CV belonged to flower length (10.18%), total soluble solids (TSS) of tuber flesh (10.44%), petal width (11.22%), flower width (12.82%), and petal length (13.44%) (Table 1).

Tuber shape showed high variation and included globose (38 accessions), oblong (34), ovate (33), obovate (40), and fusiform (40). Also, tuber skin color was highly variable, including white (50 accessions), bicolor white–violet (40), light violet (49), and dark violet (46). Tuber surface was smooth (52), bit wrinkled (79), and very wrinkled (54). Central tuber maximum transverse diameter position was predominant (112 accessions). Tuber flesh was not bitter in most of the accessions (167). Tuber flesh texture was firm in the majority of accessions (150) (Table 2). Tuber weight ranged from 1.56 to 35.70 g, tuber length varied from 21.80 to 59.15 mm, and tuber middle diameter ranged between 12.36 and 48.24 mm. Tuber flesh TSS ranged from 7.00 to 11.80%. The range of other tuber‐related characters was as follows: tuber middle diameter: 12.36–48.24 mm, tuber maximum transverse diameter: 14.20–54.63 mm, tuber collar diameter (neck): 4.79–16.86 mm, and tuber tail length: 51.76–115.22 mm (Table 1). The previous studies often stated that the turnip tuber is a taproot (Gupta et al., 2001; Lu et al., 2008; Peterson, 1973; Shattuck et al., 1991), while a few studies mentioned that the thickened part of turnip consists of both hypocotyl and root (Takahashi et al., 1994; Vogl‐Lukasser et al., 2008). The anatomical observations of Zhang et al. (2014) in six genetically diverse turnip accessions showed that turnip tubers are a combination of hypocotyl and root; both organs take part in forming the fleshy organ through secondary growth by a vascular cambium, while the proportion of hypocotyl/root differs among different accessions and seems to be independent from the geographic origin of the turnip accession. The anatomy of other tuber crops like radish (Ting & Wren, 1980), *B. napus* swede, and sugar beet resemble turnip tubers in this, while tubers from potato and kohlrabi (*B. oleracea* subsp. *gongylodes*) constitute only stem tissue, and carrot (*Daucus carota* L.) constitutes root tissue (Grubben & Denton, 2004; Huaman, 1986). Zhang et al. (2014) reported that although their study on lignification in the tubers is limited and concentrated in the first formed wood of the upper tuber parts, their observation on the presence of different degrees of lignification of the xylem from all six turnip accessions suggests that lignin biosynthesis is an important aspect of turnip tuber development.

**FIGURE 1:**
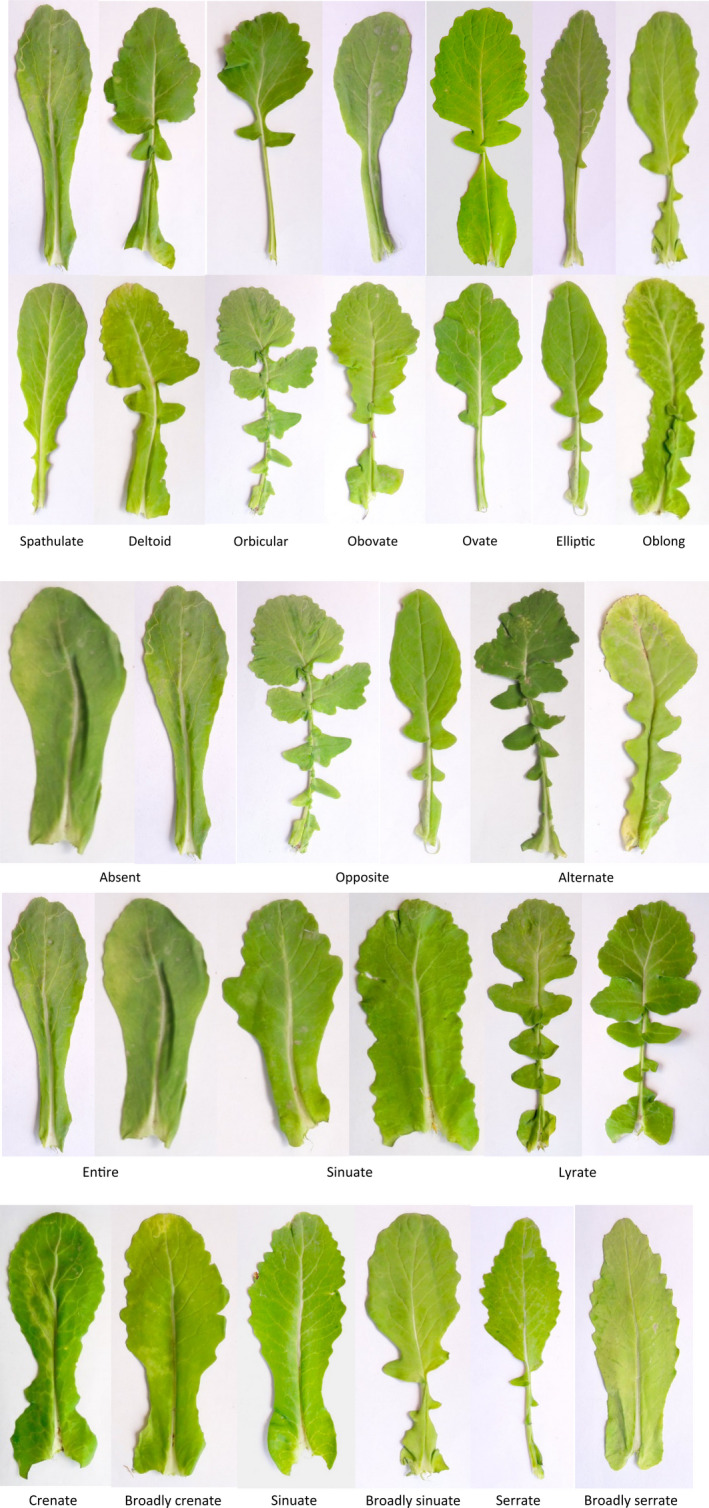
The pictures of leaves of *B. rapa* var. *rapa* accessions studied

The color of cauline leaves was light green (108 accessions) and green (77), while the color of floral leaves was strongly variable, including light green (4 accessions), green (87), dark green (87), and green–silver (7). Leaf apex shape was rounded in the majority of accessions (102). Leaf blade shape outline (lamina) was highly variable, ranging from deltoid to oblong. Flat leaf lamina was predominant (119 accessions). Leaf division was lyrate in the majority of accessions (126). Leaf margin showed strong variability, including crenate (37 accessions), broadly crenate (13), sinuate (57), broadly sinuate (55), serrate (8), and broadly serrate (15) (Table 2). The range of leaf‐related characters was as follows: leaf length: 53.00–173.20 mm, petiole length: 16.95–106.12 mm, petiole width: 1.16–4.98 mm, lamina length (lamina blade length): 28.30–99.10 mm, and lamina width (lamina blade width): 21.06–63.20 mm (Table 1). The pictures of leaves of *B. rapa* var. *rapa* accessions studied are shown in Figure 1.

Plant height until first flower ranged from 95.00 to 273.00 mm, and total plant height varied from 325.00 to 823.00 mm. Aerial part dry weight ranged from 3.20 to 252.20 g. Flowering date was predominantly in early April (162 accessions). The range of flower‐related characters was as follows: flower length: 10.75–18.62 mm, flower width: 7.80–15.21 mm, petal length: 4.95–13.14 mm, and petal width: 3.29–5.83 mm. Flowering is an important step in plant growth and defines the agriculture setting of the crop. Vernalization is the promotion of flowering after exposure to cold, where plants do not necessarily initiate flowering but acquire the competence to do so. In *B. rapa*, many investigations reported quantitative trait loci (QTL) regions or genes that regulate flowering and vernalization (Axelsson et al., 2001; Osborn et al., 1997; Teutonico & Osborn, 1995; Zhao et al., 2010). Lou et al. (2007) identified one major flowering QTL on *B. rapa* linkage group A02 that colocalized with a major turnip width QTL, using a segregating DH population from a cross between a turnip and a yellow sarson, and the BC1 from the same parents.

Silique surface outline was smooth (105 accessions), undulating (55), and constricted between seeds (65). Silique shape was cylindrical (41 accessions), spathulate (5), oblong (19), lanceolate (108), and deltoid (12). Dry silique color was highly variable, including light golden yellow (49), golden yellow (25), cream–light brown (10), light brown (86), and brown (15). Silique pedicel node color was highly variable, ranging from homochromatic to black. The range of silique number in main stem range was 2–39, while total silique number per plant was 5–820. The range of other silique‐related characters was as follows: silique length: 24.45–59.17 mm, silique width: 2.43–6.05 mm, silique thickness: 1.67–5.15 mm, silique beak length: 3.47–31.25 mm, silique pedicel length: 5.61–38.66 mm, and silique weight: 0.02–0.11 g (Table 1).

**FIGURE 2:**
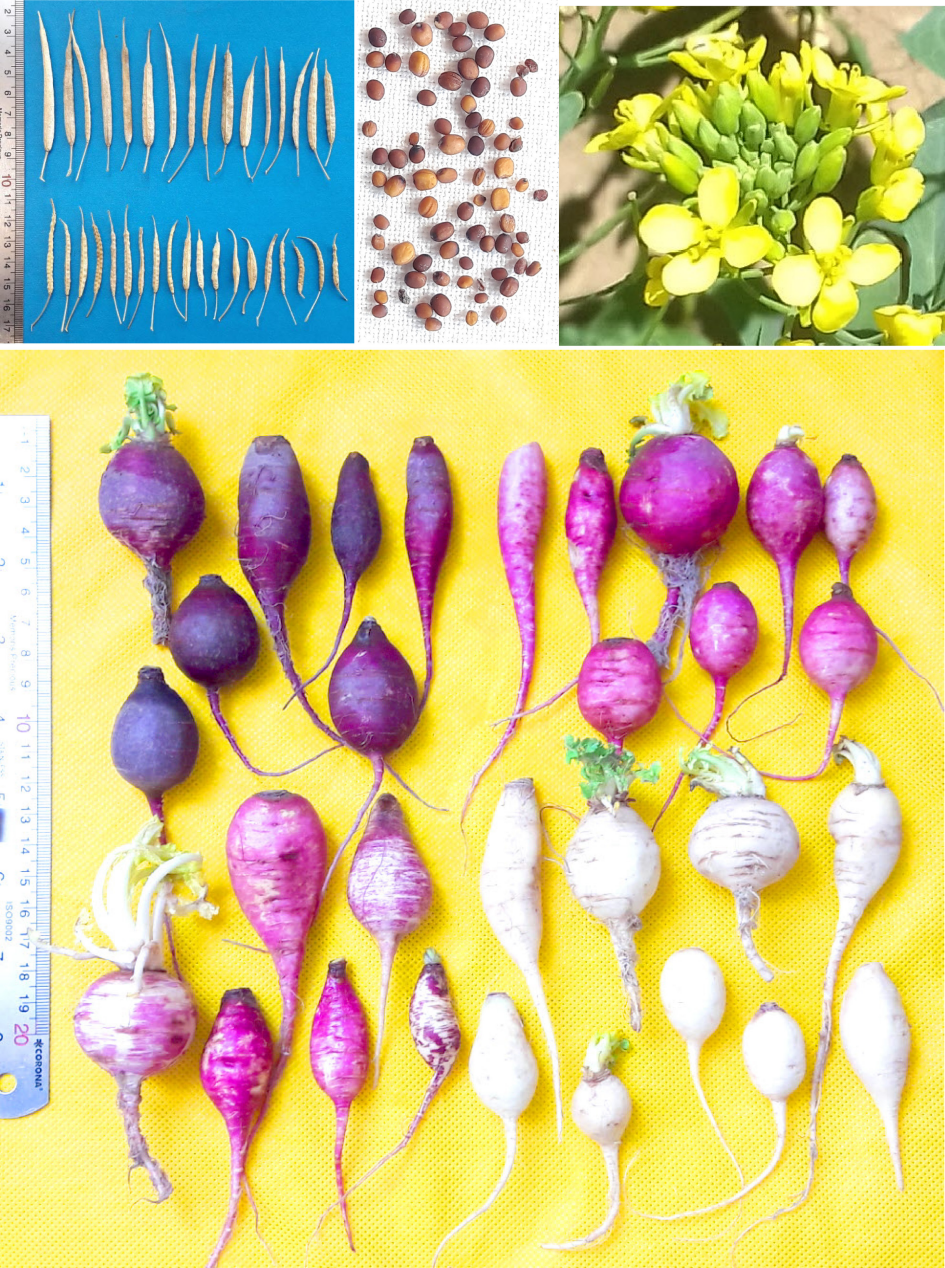
The pictures of flower, tuber, seed, and silique of *B. rapa* var. *rapa* accessions studied

Seed ripening time was in late May (99 accessions), early June (47), and mid‐June (39) (Table 2). The range of other seed‐related characters was as follows: seed length: 1.01–2.29 mm, seed width: 0.69–1.78 mm, and 1000‐seed weight: 0.00–2.16 g. Vivipary was not observed in the majority of accessions (156) (Table 1). The pictures of flower, tuber, seed, and silique of *B. rapa* var. *rapa* accessions studied are shown in Figure 2.

Pearson correlation analysis showed significant correlation between some characters (data not shown). Tuber weight showed positive correlations with tuber length (*r* = 0.73), tuber middle diameter (*r* = 0.63), tuber flesh texture (*r* = 0.51), and leaf length (*r* = 0.53).

The PCA was carried out to recognize the main differentiating characters of the variation. The eigenvalues greater than 1.00 were taken to determine which of the PC scores represented the greatest value of variability. The load values ≥0.51 were regarded as significant for each component that 18 components were extracted by explaining 74.88% of total variance (Table 3).

**TABLE 3:** Eigenvalues of the principal component axes from the PCA of the morphological characters in the studied *B. rapa* var. *rapa* accessions

Character	Component																	
	1	2	3	4	5	6	7	8	9	10	11	12	13	14	15	16	17	18
Tuber shape	−0.25	0.44	−0.03	0.18	0.12	−0.07	0.04	−0.07	0.06	0.63**	−0.19	−0.06	0.13	0.11	−0.07	−0.05	0.24	−0.14
Tuber skin color	−0.08	−0.08	−0.04	0.15	−0.05	−0.06	−0.01	0.60**	0.04	−0.08	0.10	0.16	0.13	0.48	−0.15	0.30	−0.08	−0.10
Tuber weight	0.92**	−0.01	0.11	−0.04	−0.09	0.09	−0.02	0.01	0.06	0.01	0.08	0.00	0.05	−0.05	0.06	−0.05	−0.05	0.01
Tuber surface	0.41	−0.09	0.17	−0.04	0.05	0.05	−0.02	0.09	0.15	−0.08	−0.12	0.13	−0.23	0.51**	−0.07	0.02	−0.14	−0.02
Tuber length	0.34	0.27	0.05	0.05	−0.02	−0.04	0.11	0.28	0.08	0.17	−0.07	0.00	0.47	0.22	0.01	0.18	0.31	0.01
Tuber middle diameter	0.91**	−0.09	0.04	0.04	−0.07	0.06	−0.07	0.12	0.12	0.01	0.05	0.06	0.05	0.05	0.01	−0.02	−0.10	0.01
Tuber maximum transverse diameter	0.88**	−0.04	0.11	0.09	0.02	0.14	−0.04	0.05	0.18	0.06	−0.05	−0.05	0.01	0.12	−0.01	0.06	−0.13	−0.04
Tuber maximum transverse diameter position	0.22	0.09	−0.10	−0.07	0.19	−0.32	0.25	0.47	0.00	0.46	−0.19	−0.06	0.03	−0.19	0.06	−0.06	−0.13	0.07
Tuber collar diameter (neck)	0.70**	−0.05	0.29	0.00	−0.15	0.04	0.04	−0.22	0.11	−0.11	0.04	−0.01	0.06	−0.13	0.08	−0.18	0.08	0.04
Tuber tail length	0.38	0.02	0.03	0.02	−0.08	0.08	−0.02	−0.08	0.09	−0.05	−0.04	−0.04	0.57**	−0.02	−0.01	0.16	0.07	0.13
Tuber flesh bitterness	0.18	0.04	0.09	−0.23	−0.09	0.04	−0.01	0.00	−0.18	−0.13	0.13	−0.29	−0.32	−0.40	0.24	−0.08	0.04	0.32
Tuber flesh texture	0.09	−0.03	−0.18	0.09	0.10	0.08	0.07	0.38	−0.09	0.14	0.00	0.07	0.07	0.03	−0.16	0.26	−0.18	0.16
Tuber flesh TSS	−0.07	−0.40	−0.05	0.19	0.00	0.13	−0.07	−0.01	−0.07	−0.05	−0.02	−0.16	0.62**	0.00	−0.23	0.00	0.02	−0.18
Tuber main shoot number	0.36	−0.07	0.10	0.21	0.11	−0.12	0.18	0.08	−0.04	−0.20	−0.01	0.47	−0.14	−0.32	−0.01	0.16	0.05	0.06
Leaf length	−0.16	0.70**	0.22	0.10	0.11	0.03	0.07	−0.02	0.08	0.31	−0.03	0.35	−0.07	−0.10	0.17	0.14	0.06	0.00
Petiole length	−0.25	0.37	0.18	0.23	0.06	0.12	−0.14	0.06	0.13	0.36	0.05	0.38	−0.09	−0.21	0.16	0.16	0.17	−0.03
Petiole width	−0.08	0.83**	0.00	0.06	0.07	0.13	−0.06	−0.07	0.03	−0.04	−0.11	−0.10	0.01	−0.07	−0.12	−0.10	0.09	−0.13
Clasping of stem by upper leaves	0.02	0.14	0.19	−0.13	0.03	0.04	−0.01	0.25	0.26	−0.14	−0.31	0.03	−0.03	−0.48	0.01	−0.08	−0.11	0.01
Cauline leaves color	−0.01	0.14	0.17	−0.10	0.04	0.12	0.20	0.05	−0.08	0.05	−0.03	0.64**	0.03	0.05	0.38	0.06	−0.15	−0.11
Floral leaves color	0.06	0.26	0.17	0.15	0.14	−0.04	0.03	0.04	0.08	0.67**	−0.16	0.32	−0.12	0.04	−0.11	0.01	−0.10	−0.01
Lamina length (lamina blade length)	0.09	0.68**	0.15	−0.11	0.14	−0.09	0.28	−0.17	−0.03	0.06	−0.14	0.14	−0.02	0.14	0.10	0.01	−0.04	0.04
Lamina width (lamina blade width)	−0.05	0.76**	0.11	0.14	−0.02	0.07	−0.12	0.07	0.03	0.13	0.09	0.03	−0.04	−0.08	−0.01	0.10	−0.03	0.00
Leaf apex shape	−0.05	−0.13	−0.07	−0.12	0.11	0.05	−0.11	−0.12	−0.17	−0.10	0.09	−0.63**	0.07	−0.19	0.30	0.03	0.09	0.02
Leaf blade shape outline (lamina)	−0.04	−0.01	−0.27	−0.44	0.07	0.07	0.02	−0.04	0.08	0.05	0.00	−0.09	0.04	0.13	0.21	0.00	0.56**	0.06
Leaf lamina attitude	0.05	−0.22	0.17	0.13	0.04	−0.02	0.21	−0.09	0.23	−0.03	0.00	−0.02	−0.03	0.11	0.65**	0.05	0.05	0.10
Leaf lamina blistering	0.09	0.25	0.03	0.04	0.08	0.05	−0.13	−0.16	−0.02	−0.02	−0.12	0.00	−0.07	−0.20	0.66**	−0.10	0.01	−0.09
Leaf division	−0.10	0.15	0.10	0.86**	0.04	0.04	−0.06	−0.05	0.04	0.15	0.07	0.01	−0.02	0.12	0.13	0.00	0.10	0.03
Leaf margin	0.06	−0.32	−0.17	−0.37	−0.08	0.12	0.06	0.14	−0.15	−0.50	0.22	0.29	0.06	0.08	−0.16	0.18	0.11	0.08
Leaf lobelet number	0.14	0.02	−0.01	0.83**	0.02	0.16	−0.04	0.01	0.00	0.20	−0.01	0.12	0.03	−0.06	0.12	−0.12	−0.03	−0.08
Lobelet arrangement	0.08	0.03	0.09	0.81**	0.04	−0.14	−0.03	0.15	0.06	−0.08	0.07	0.00	0.12	0.08	−0.11	0.11	−0.03	0.04
Flowering date	−0.07	−0.01	−0.06	0.30	0.19	0.38	−0.19	−0.33	−0.01	−0.18	0.07	−0.02	0.03	0.42	0.04	0.16	−0.01	0.16
Plant height until first flower	0.39	0.08	0.54**	0.20	−0.04	−0.04	0.01	−0.15	0.06	0.20	−0.06	0.16	−0.30	−0.12	0.11	0.10	−0.14	0.16
Total plant height	−0.01	0.30	0.46	0.24	0.05	0.15	0.10	0.09	−0.04	0.28	−0.04	0.12	0.15	0.23	0.15	−0.29	−0.04	0.05
Flowering branch number	0.20	0.17	0.79**	−0.09	−0.02	0.22	0.05	0.06	−0.01	−0.08	0.07	0.05	0.08	−0.07	0.01	−0.12	−0.04	0.01
Leaf number until first flower	0.17	−0.03	0.82**	0.09	0.07	0.15	−0.06	−0.20	0.09	0.04	−0.06	−0.01	−0.10	−0.02	0.10	0.06	0.11	0.02
Total leaf number	0.08	0.17	0.84**	0.14	0.02	0.25	0.05	−0.02	0.03	0.09	−0.01	0.10	0.04	0.03	0.01	0.08	−0.02	0.00
Flower length	−0.01	0.00	0.01	0.03	0.86**	−0.02	0.11	0.12	0.05	0.18	−0.09	0.08	−0.05	0.06	0.06	0.20	0.06	0.03
Flower width	−0.05	0.01	−0.03	0.08	0.83**	0.08	0.11	0.07	0.09	0.07	−0.10	−0.01	−0.08	0.01	0.04	−0.03	0.04	0.10
Petal length	−0.02	0.08	0.10	0.01	0.83**	−0.06	0.07	0.03	0.06	0.02	−0.07	−0.03	−0.01	−0.03	0.02	0.05	0.08	−0.01
Petal width	−0.20	0.16	−0.03	−0.05	0.71**	−0.07	−0.12	−0.01	0.15	−0.09	0.13	−0.04	0.07	0.01	−0.01	−0.17	−0.15	−0.10
Seed ripening time	−0.21	0.15	−0.05	−0.10	−0.03	0.41	0.29	0.15	−0.08	−0.49	0.12	0.05	0.28	−0.01	0.04	0.16	−0.04	0.16
Silique number in main stem	0.18	0.02	0.26	0.12	−0.08	0.75**	0.12	−0.05	0.14	−0.11	0.15	−0.08	−0.08	0.07	0.00	0.00	0.04	0.04
Total silique number in plant	0.14	0.07	0.16	0.01	−0.02	0.89**	0.09	0.03	0.00	−0.03	0.02	0.00	0.03	−0.03	0.02	0.03	0.01	0.02
Silique length	0.13	0.18	−0.05	0.02	0.09	0.06	0.04	0.12	0.75**	0.02	0.10	0.03	0.06	0.12	0.16	0.12	−0.02	0.04
Silique width	−0.03	0.03	0.06	−0.06	0.15	0.09	0.80**	−0.10	0.17	0.18	0.10	−0.03	−0.19	0.08	0.04	−0.11	0.12	0.13
Silique thickness	−0.04	0.02	−0.02	−0.03	0.06	0.08	0.80**	0.09	0.14	0.01	0.00	0.19	0.00	−0.01	−0.01	0.16	0.00	0.00
Silique surface outline	0.15	0.19	0.00	0.22	−0.14	−0.33	−0.58**	−0.09	0.14	0.05	−0.18	−0.05	−0.27	0.08	0.08	0.06	0.01	−0.02
Silique shape	0.19	0.03	0.07	−0.11	0.15	0.06	0.22	−0.20	0.08	0.19	0.05	−0.25	−0.47	0.27	−0.28	0.09	0.26	0.19
Dry silique color	0.05	−0.13	0.05	−0.04	0.04	0.27	0.45	−0.09	−0.26	−0.28	−0.08	0.21	0.01	−0.15	−0.11	0.35	0.17	−0.16
Silique shattering	0.18	−0.05	−0.08	−0.09	−0.03	0.01	−0.10	0.02	0.02	0.04	−0.09	0.06	−0.03	0.08	0.03	0.00	−0.73**	−0.02
Silique beak length	0.21	−0.22	0.10	−0.10	0.13	0.00	0.23	−0.05	0.34	0.12	−0.07	−0.26	−0.27	−0.11	0.10	0.37	−0.14	−0.20
Silique pedicel length	−0.12	0.14	−0.01	0.03	0.07	0.08	0.07	0.17	0.11	−0.08	−0.05	0.10	0.14	0.13	−0.04	0.68**	0.05	−0.07
Distinct silique pedicel node color	−0.05	−0.10	−0.01	−0.08	0.14	0.07	0.02	0.88**	0.04	−0.04	−0.02	−0.01	−0.05	−0.14	0.00	−0.04	0.06	−0.01
Silique pedicel node color	0.05	0.00	−0.06	0.11	0.03	0.05	−0.01	0.89**	−0.08	−0.04	−0.07	0.08	0.00	0.02	−0.09	0.05	0.00	−0.09
Silique weight	0.06	0.14	0.04	0.11	−0.09	0.03	0.59**	0.14	0.40	−0.31	0.09	0.00	−0.02	−0.10	0.17	0.03	0.07	−0.22
Silique total seed number (rounded +wrinkled)	0.12	0.01	0.01	0.01	0.15	0.04	0.15	−0.05	0.78**	0.17	−0.10	0.04	0.00	−0.03	−0.09	0.05	0.14	0.11
Silique seed number (rounded)	0.24	−0.08	0.14	0.07	0.12	0.01	0.09	−0.15	0.74**	−0.03	−0.04	0.04	−0.01	−0.06	0.04	−0.07	−0.08	0.15
Seed color	0.01	−0.11	0.08	−0.01	0.05	0.09	0.01	−0.08	0.27	−0.07	−0.15	−0.06	−0.01	−0.03	−0.02	−0.06	0.05	0.78**
Seed length	−0.05	−0.01	−0.04	−0.04	−0.10	0.06	0.35	0.04	−0.08	−0.20	0.77**	−0.02	0.00	0.05	0.05	0.03	0.07	−0.12
Seed width	0.01	−0.09	0.03	0.17	0.01	0.07	0.14	−0.04	0.03	−0.04	0.82**	−0.14	−0.09	−0.06	−0.05	−0.14	0.14	−0.19
1000‐seed weight	0.15	−0.05	0.00	−0.02	−0.12	0.15	−0.25	−0.10	0.02	−0.09	0.74**	0.06	0.04	0.06	−0.10	0.06	−0.12	0.16
Vivipary	0.01	−0.04	0.09	−0.18	−0.36	−0.02	−0.04	−0.12	0.04	−0.05	0.15	−0.09	0.39	0.04	0.10	0.42	−0.32	0.20
Aerial part dry weight	0.06	0.05	0.19	−0.06	0.01	0.88**	0.12	0.10	0.02	0.02	0.07	0.06	0.09	−0.01	0.02	0.03	−0.03	0.02
Total	4.47	3.61	3.28	3.25	3.22	3.20	3.09	3.08	2.67	2.61	2.43	2.06	2.04	1.80	1.73	1.66	1.63	1.33
% of Variance	7.09	5.74	5.21	5.15	5.11	5.08	4.91	4.89	4.24	4.15	3.86	3.26	3.24	2.86	2.75	2.64	2.59	2.11
Cumulative %	7.09	12.83	18.04	23.20	28.30	33.39	38.30	43.18	47.42	51.57	55.43	58.70	61.93	64.79	67.54	70.18	72.77	74.88

Note

**Eigenvalues ≥0.51 are significant at the *p* ≤ .01 level.

Four tuber‐related traits, including tuber weight (0.92), tuber middle diameter (0.91), tuber maximum transverse diameter (0.88), and tuber collar diameter (neck) (0.70) with positive correlations were found to be influential in PC1. The PC2 was correlated with four leaf‐related traits, including leaf length (0.70), petiole width (0.83), lamina length (lamina blade length) (0.68), and lamina width (lamina blade width) (0.76) with positive correlations. The PC3 was correlated with plant height until first flower (0.54), flowering branch number (0.79), leaf number until first flower (0.82), and total leaf number (0.84). The remaining characters loaded significantly in the rest components (PC4–PC18) and explained less variability.

**FIGURE 3:**
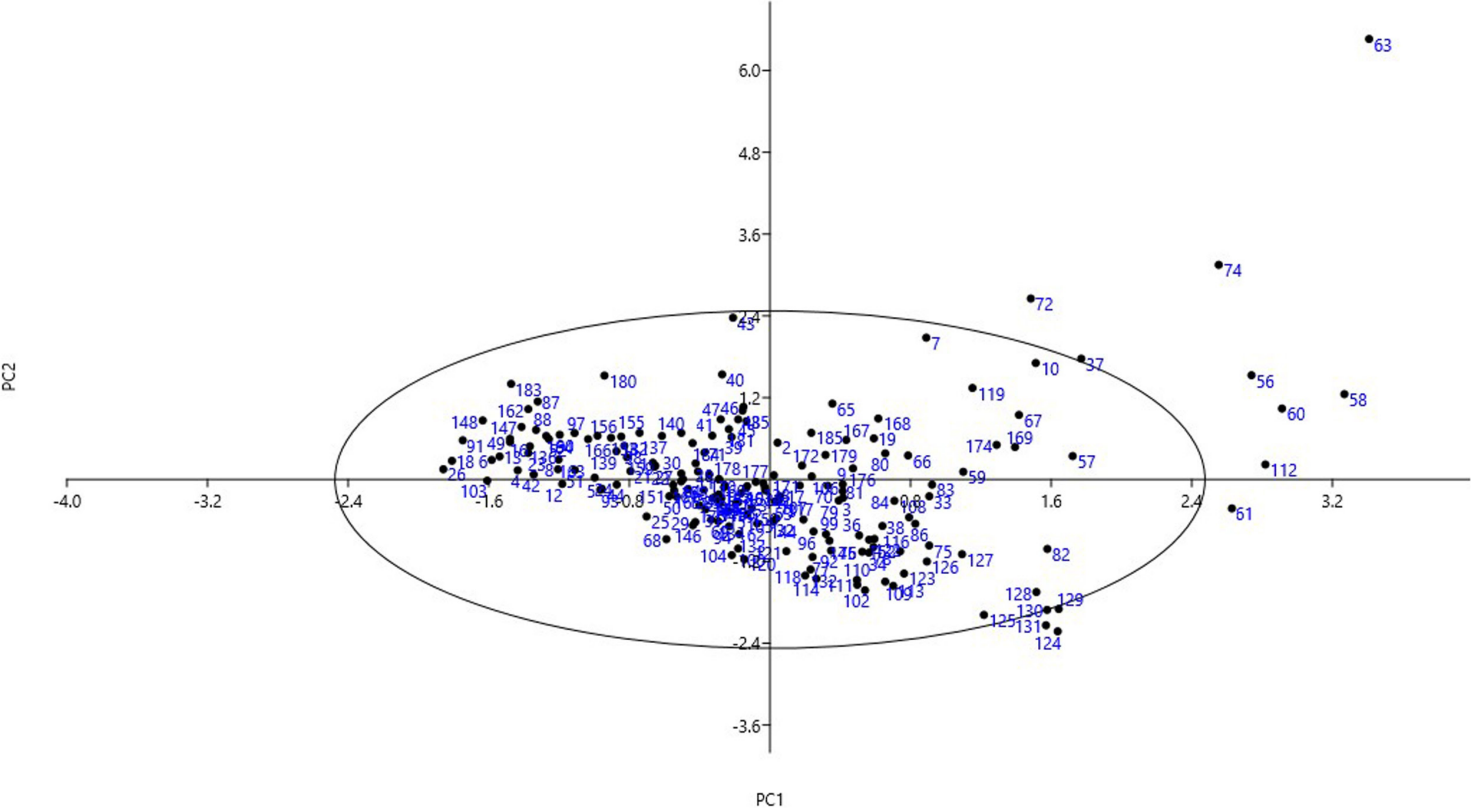
Scatter plot for the studied *B. rapa* var. *rapa* accessions based on PC1/PC2

The scatter plot created based on the PC1 and PC2 (Figure 3) showed that the accessions with close proximity were more similar in terms of effective traits in PC1 and PC2 and were placed in the same group. The scatter plot showed that residuals of the majority of accessions bounce randomly around 0.00 line forming the horizontal band. This suggests that the variances of the error terms are equal and the relationship among the accessions is linear. However, 10 outliers were observed among the accessions evaluated, which might be due to their extreme values for particular traits.

**FIGURE 4:**
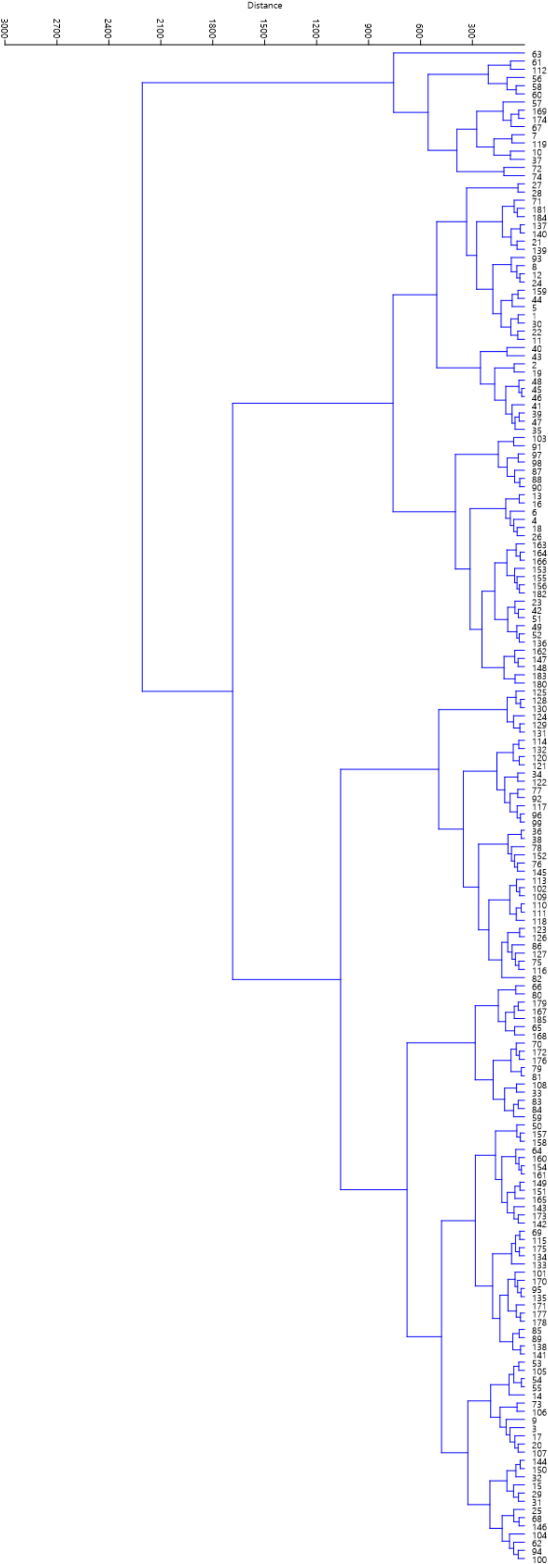
Ward cluster analysis of the studied *B. rapa* var. *rapa* accessions based on morphological traits using Euclidean distances

The dendrogram obtained based on all the characters measured clustered the accessions into two major clusters (Figure 4). Sixteen accessions were placed into the first cluster. The rest of the accessions were placed into the second cluster, which was divided into six subclusters, indicating high variability among the accessions.

Here, a high level of morphological variabilities was observed among the accessions, which is applicable and useful for *B. rapa* var. *rapa* breeding programs. Matthaus et al. (2016) reported high diversity among the canola (*Brassica napus* L.) cultivars from Turkey. Agro‐morphological‐based variation is important to screen best accessions in field experiment. The diverse agro‐morphological‐based accessions are useful for further biochemical and molecular evaluation. Genetic diversity study is used for efficient utilization and for development of improved cultivar/varieties (Jan et al., 2016; Shinwari et al., 2014). Morphological‐based screening of different crop species/subspecies is therefore so much important for all plant breeders (Iqbal et al., 2015; Martins et al., 2006). Therefore, proper strategies and planning is needed to evaluate local and exotic germplasm and to screen best accessions among these for both qualitative and quantitative characters (Balkaya & Ergun, 2008). The conservation strategies are important for many reasons as it is best source to conserve threaten/endangered species, for further crop improvement through new morphological techniques, and to develop new breeding cultivars/varieties (Baranger et al., 2004).

## 4 CONCLUSION

Estimation of different qualitative and quantitative traits offers a distinct means to improve varied accessions. Significant variations were recorded for various morphological traits among the accessions of *B. rapa* var. *rapa*. At the present time, breeding programs, particularly with an obligate outcrossing crop such as turnip, present a challenge. Based on the commercial and quality traits, 17 accessions, including no. 40, 72, 17, 10, 7, 19, 112, 98, 83, 2, 4, 1, 5, 9, 37, 6, and 8, could be selected for direct cultivation. Also, the promising accessions identified here can be utilized directly in breeding programs for genetic enhancement of this crop.

## CONFLICT OF INTEREST

The authors declare no conflict of interest.

## RESEARCH INVOLVING HUMAN PARTICIPANTS AND/OR ANIMALS

None.

## INFORMED CONSENT

None.

## Data Availability

The data that support the findings of this study are available from the corresponding author upon reasonable request.
